# Well-Being of Adolescents in De-Escalation Situation: Physical, Emotional, Social, and Academic Impact

**DOI:** 10.3389/fpsyg.2021.646027

**Published:** 2021-08-25

**Authors:** Naiara Berasategi Santxo, Nahia Idoiaga Mondragon, Naiara Ozamiz-Etxebarria, Maria Dosil-Santamaria

**Affiliations:** ^1^Department of Didactics and School Organization, University of the Basque Country, Leioa, Spain; ^2^Department of Developmental and Educational Psychology, University of the Basque Country, Leioa, Spain; ^3^Department of Research and Diagnostic Methods in Education, University of the Basque Country, Leioa, Spain

**Keywords:** well-being, school task, emotion, adolescence, COVID-19 pandemic

## Abstract

The Covid-19 pandemic has changed the world we knew in recent months. In the interest of maintaining social distance, lockdown periods have been established and schools in many countries have closed their doors. In this context, the objective of this research was to analyze the well-being of adolescents in Spain after lock-down and during the de-escalation process in a holistic way; taking into account their indicators on physical, emotional, social, and academic levels. The “Well-being of Children in Lockdown” (WCL) scale was used to measure the well-being of adolescents using these same parameters. The results point out that the general well-being of adolescents in the pandemic situation was situated at an intermediate level. Taking into account the different aspects measured within the general well-being, the domains that obtained the lowest scores were the domains of addictions and playful and creative activities. Intermediate scores were also obtained in the physical activity, emotional and academic domains, with the routine and academic domains having the highest scores. Boys and younger adolescents are those who show higher scores in the general well-being. Moreover, correlations appear between academic task stress and emotions, playful and creative activities, addictions, physical activity, routine, academic and overall well-being.

## Introduction

The COVID-19 pandemic has changed the world as we knew it in few months. Although the first cases of an unknown pneumonia appeared at the end of 2019 in the city of Wuhan (China), its expansion was exponential, affecting China first and then spreading to the rest of the world (Chen Q. et al., 2020; Torales et al., 2020). In fact, since the beginning of March, COVID-19 has been considered a pandemic and countries world-wide have created contingency plans to face it (WHO, 2020).

In the interest of reducing COVID-19 infections, social distancing has been a crucial tool. In consequence, schools in many countries closed their doors and adolescents were confined to their homes all over the world (United Nations Educational, Scientific and Cultural Organization, 2020). However, the management of such lockdowns or their restrictions varied in each country. Spain was one of the countries with the hardest restrictions for children and adolescents, as for 6 weeks (from March 14 to April 26) they were completely locked down and forbidden to leave their homes (Garcia, 2020). From April 26 onward, the Spanish government allowed children under 14 to go out, but only for 1 h every day and always near their homes (Lucas, 2020). As of May 4, people over 14 were also allowed to go out, but only to practice sports and this also with time restrictions. When this research was carried out, children under 14 could go out of their houses only 1 h a day, while those over 14 years of age were able to go out for more time slots (Sanchez, 2020).

It is crucial to analyze the disruptive effects of COVID-19 on adolescents as this could have multiple consequences in their lives; they are a vulnerable group who are experiencing a difficult time of transition (Larsen and Luna, 2018). The consequences may include chronic and acute stress, concern for their families, unexpected life losses, or increased access to the Internet and social networks due to the abrupt interruption of classes and lockdown (Guessoum et al., 2020). In fact, the consequences that this period of lockdown may have on children and adolescents have aroused the concern of pediatricians, psychologists, and educators (Grechyna, 2020; Jiloha, 2020). These professionals point out that the well-being and the health of adolescents could be affected at different levels and that it is essential to investigate this in order to help them to better cope with this crisis (Dalton et al., 2020; Wang et al., 2020).

Therefore, as far as age is concerned, it is well known that adolescence represents a critical period of rapid physical, social, cognitive, and emotional changes, with important implications for health and well-being in later life (Pigaiani et al., 2020). Different studies around the world have shown that younger age has been where people have shown less well-being during the pandemic (Pieh et al., 2020). However, few studies have been conducted to indicate at what point in adolescence may have had the greatest influence on people’s well-being.

But in the few studies that have been conducted, it has been shown that among adolescents, being in the higher grades is a risk factor for adolescents (Chen S. et al., 2020; Zhou et al., 2020). This is because the higher the academic course the greater the pressure on students (Zhang et al., 2005; Yuan et al., 2018) and because their independence is more compromised. In addition, the older is the adolescent, the greater is the importance of social relationship. Adolescents have amplified energy, novelty, motivation, curiosity, and enthusiasm that make it difficult to isolate them at home. The hormonal changes that accompany puberty combine with the social dynamics of adolescents to make them very sensitive to social status, peer group, and relationships. Adolescents may feel frustrated, nervous, disconnected, homesick, and bored due to social distancing during this pandemic (Imran et al., 2020).

In terms of gender, both studies conducted worldwide with adults (Moghanibashi-Mansourieh, 2020; Pieh et al., 2020; Rossi et al., 2020) and adolescents (Chen S. et al., 2020; Engel de Abreu et al., 2021), point to females having worse well-being during the COVID-19 pandemic. This is because there is still much to be done for gender equality, which is what increases women’s well-being (Diener et al., 2018).

Although the concept of well-being is highly variable and has been studied across a wide range of disciplines, age groups, cultures, communities, and countries, resulting in an assortment of definitions (Pollard and Lee, 2003), from a holistic point of view, well-being has been defined as “a multidimensional construct incorporating mental/psychological, physical and social dimensions” (Columbo, 1986, p. 288). In the same vein, it should be borne in mind that, according to the World Health Organization (WHO, 1946), “Health is a state of complete physical, mental and social well-being and not merely the absence of disease or infirmity” (p. 100).

In the case of adolescents, school closures and social distancing could be even more harmful to their well-being since during adolescence young people grow in independence and give priority to their peers over their parents. The disruption of these peer-to-peer direct contacts may therefore pose significant challenges to young people’s well-being (The Lancet Child Adolescent Health, 2020).

On the physical level, it has been already demonstrated that lockdown has brought about a decrease in physical activity accompanied by an increase in sedentary behavior (Ammar et al., 2020). A study conducted in Latin America and Europe found a high prevalence of physical inactivity among adolescents during lockdown, regardless of their country. Consumption of ultra-processed foods was also high during this period in both regions (Ruíz-Roso et al., 2020). The lack of activity and poor nutrition that can occur in a COVID-19 lockdown may have the effect of causing or worsening obesity among adolescents. This is an issue to be aware of, since obesity can worsen the prognosis of adolescents who become infected with COVID-19 (Nogueira-de-Almeida et al., 2020).

On the emotional level, the rapid increase in the number of cases of infection and deaths, disruption of daily routines, lockdown, fear of infection, social distancing from peers and friends, and lack of access to educational resources may create feelings of uncertainty and anxiety among adolescents (Imran et al., 2020). Adolescents may also experience anxiety as they try to understand the pandemic and the threat it poses to them, their families and friends (The Lancet Child Adolescent Health, 2020). A study carried out during the lockdown in the Netherlands collected the subjective experience of the COVID-19 pandemic among adolescents (Janssen et al., 2020). Results highlighted the fact that they were disturbed by the lack of social contact with friends, irritated with their family members and concerned about the health of others.

On the social level, social distancing and school closures are likely to result in greater feelings of loneliness for adolescents (Loades et al., 2020). Many young people have been concerned about losing connection with friends, non-immediate family, and other trusted adults. This may be especially dramatic among those vulnerable adolescents who did not feel safe to talk with others from their homes or who have limited access to technology. In addition, some young people have already pointed out that they felt that talking online is not as satisfying as being in direct contact with their friends (Young Minds, 2020). In fact, from the beginning of the lockdown, more than one third of the adolescents reported high levels of loneliness (Oxford ARC Study, 2020; Young Minds, 2020). This feeling of loneliness may be due to the importance of peers in the evolutionary stage of adolescence (Meeus and Dekoviíc, 1995).

On the academic level, school and university closures had affected over 91% of the world’s student population (Lee, 2020). This may have important consequences as schools create structures in the routines of the adolescents that help their emotional stability (Young Minds, 2020). Moreover, the closure of schools has created great uncertainty among adolescents about their future, causing anxiety because of the cancellation of exams, exchange programs, and academic events (Lee, 2020). For teens in vulnerable or at-risk situations, school closures can also represent the loss of a stable and safe environment.

In sum, the COVID-19 pandemic is creating multiple threats to adolescents’ health and well-being. For this reason, it is necessary to conduct studies that determine how they are coping both with lockdown and the de-escalation processes or periods of restrictions. Moreover, adolescents’ well-being should not be researched from a single perspective, level or indicator, as their lives are lived in multiple domains and each domain influences their well-being (Ben-Arieh et al., 2001; Bradshaw and Mayhew, 2005; Hanafin et al., 2007; Land et al., 2007).

In this context, the objective of this research is to analyze the well-being of adolescents in the pandemic situation. In fact, the contextualization of this study goes beyond the period of lockdown and aims equally to study the welfare of adolescents in periods of restrictions due to COVID-19 in the north of Spain. Moreover, their well-being is researched in a holistic way, that is, taking into account their indicators at physical, emotional, social, and academic levels or domains. Moreover, whether well-being is affected by the age or gender and by stress related to academic tasks is also analyzed.

## Methodology

### Sample

The sample was comprised of 500 adolescents from the Basque Autonomous Country (North of Spain). Of the sample, 64.6% were girls (*n* = 323), 34.8% were boys (*n* = 174) and.6% non-binary (*n* = 3). With regard to age, 41.7% were aged between 12 and 14 years (*n* = 207), 35.5% between 15 and 16 years (*n* = 176) and 22.8% between 17 and 18 years (*n* = 113). Of those%15.2 were attending to the first course of secondary education (*n* = 76), 14.6% second course of secondary education (*n* = 73), 24% third grade of secondary education (*n* = 120), 17.2% fourth grade of secondary education (*n* = 86), 10% first grade of bachelor studies (*n* = 50), 14% second grade of bachelor studies (*n* = 70), 2.2% first grade of professional training (*n* = 11) and 0.6% second grade of professional training (*n* = 3). According to their family socio-economic status the majority 42.3% has a bachelor studies (*n* = 211.5) or 28.7% university studies (*n* = 143.5), 15.1% has secondary studies (*n* = 75.5) and 12.1% primary studies (*n* = 60.5) and 1.9% do not have studies (*n* = 9.5). Regarding to their parents occupation the majority 37.5 was specialized worker (such as plumber, waiter, etc.), 29.7% were has high degree (such as layer, teacher, doctor, etc.) (*n* = 148.5), 8% were manufacture worker or laborer (*n* = 49), 7.2% is the owner of a big company (with more than 25 employees) (*n* = 36), 3.4% was retired (*n* = 17), 2.3% were unemployed (*n* = 11.5%) and 1.9% was domestic worker.

### Instrument

The “Well-Being of Children in Lockdown (WCL)” scale (Berasategi et al., 2020) was employed to measure the well-being of adolescents at physical, emotional, social, and academic levels. This scale had been previously validated for the Spanish child population (Berasategi et al., 2020). It consists of 22 items and the overall alpha value of the scale was 0.804. The instrument comprises six sub-scales: emotions (five items), playful and creative activities (four items), academic (three items), addictions (four items), routine (four items), and physical activity (two items). The items included in the first domain are related to emotions (such as crying more than usual, feeling more nervous than usual, getting angry more than usual); those in the second are related to playful or creative activities (such as taking part in creative activities; e.g., theater, music, and art, playing different games throughout the day, doing leisure activities with family throughout the day); the third domain consists of items related to academic issues (such as having been sent materials, assignments, and homework by your school, spending enough time on your schoolwork during the day, working on school projects with your family throughout the day); the items of the fourth are related to addictive habits regarding technology or eating [such as eating more treats (e.g., cookies, chocolate, and chips) during lockdown, over-using new technology]; the fifth domain is concerned with daily routines including the maintenance of a daily schedule of eating and sleeping habits (such as having an agreed routine and trying to stick to it, eating a well-balanced diet, having healthy sleeping habits); and finally, the sixth domain contains items related to physical activity (such as getting enough physical exercise during the day, moving their body around sufficiently). The participants were required to respond on a 4-point Likert-type scale ranging from strongly agree (4) to strongly disagree (1). The data from the negative statements were recoded positively. Therefore, in the domain of emotions, you will measure how many positive emotions are felt and the domain of addictions will be higher the fewer addictions they have.

The estimated reliability coefficients were for the original scale 0.872 for the first factor, 0.783 for the second, 0.696 for the third, 0.627 for the fourth, 0.646 for the fifth, and 0.847 for the sixth factor. The reliability of the entire scale was 0.804. The various domains and the overall scale have shown adequate values of internal consistency (>0.60). In this case scale was administrated to a population between 15 and 18 years and also the reliabity coeficients shown adecuate internal consistency, for the first factor 0.813, for the second 0.806, 0.676 for the third, 0.645 for the fourth, 0.707 for the fifth, and 0.605 for the sixth factor. The reliability of the entire scale was 0.787. The various domains and the overall scale have shown adequate values of internal consistency (>0.60).

In addition, four *ad hoc* questions were asked specifically for this research and related to the stress of academic work during confinement. The responses for these three items were Likert-type from 1 to 4 points, from strongly agree (4) to strongly disagree (1). The items were “I am happy to be at home and do homework from home,” “Homework is a source of conflict at home,” “I feel that I am stressed by the large amount of homework,” “I spend too much time doing homework from home.” The reliability of the items as a whole was 0.85. Sociodemographic data were also collected, namely the sex and age of the adolescents.

### Procedure

In order to recruit the participants, all the centers registered in the database of the Department of Education of the Basque Government were contacted by email and were asked to disseminate an online questionnaire to their students. Both the data of the sample and the parents’ consent for participating in the study were collected with the help of Google online forms. Family members were informed of the research study by e-mail. In the same questionnaire, it was explained that participation in the study was voluntary and anonymous. Moreover, the parents or legal guardians of the children gave written consent for two phases of this research. First, consent was given to analyze the data and second, to make the data public in scientific articles while respecting anonymity. A total of 30 questionnaires were excluded for not giving consent for this second phase. The data were collected during the first phase of the de-escalation, May 11–25, 2020. This research has obtained the approval of the Ethics Committee of the UPV/EHU [M10/2020/055].

### Data Analysis

Data were analyzed using IBM SPSS Statistics for Windows, Version 26.0, Armonk, NY, United States. The ANOVAs were used to analyze the difference between gender and age. To analyze differences between categoric variables, the Tuckey statistic was used. In turn, the effect size associated with each comparison of means was calculated by using the square eta. The Pearson *r* statistic was used to made correlations between the different variables analyzed.

## Results

### The Well-Being of Adolescents From a Holistic Perspective

The overall levels of well-being of adolescents are in an intermediate level under 3 (*M* = 2.68) (see Table 1). Likewise, the media of the different domains measured by the scale are also between 2 and 3. The lowest scores are obtained in the practice of playful and creative activities (*M* = 2.18), addictions (*M* = 2.34), and physical activity (*M* = 2.59) domains. The highest scores are obtained in routine (*M* = 3.03), with this being the only domain that is higher than three points, and in the academic domain (*M* = 2.97).

**TABLE 1:** Descriptive analysis of the different dimensions of well-being.

Dimensions	*n*	Min	Max.	*M*	SD
Emotions	500	1.67	4.00	2.79	0.71
Playful and creative activities	500	1.00	4.00	2.18	0.39
Academic	500	1.00	4.00	2.97	0.46
Addictions	500	1.00	4.00	2.34	0.56
Routine	500	1.00	4.00	3.03	0.57
Physical activity	500	1.00	4.00	2.59	0.73
Well-being Total	500	1.52	3.81	2.68	0.36

Taking into account the responses (see Table 2) in the academic domain, 68% (*n* = 340) of the adolescents think that they have received too many academic tasks and 40.8% (*n* = 204) think that they have spent too much time on these. However, only 5.2% (*n* = 26) said that they spent a lot of time doing school projects with their parents.

**TABLE 2:** Percentages of well-being of their children in confinement situation: academic aspect, routine, physical activity, emotions, additions, playful, and creative activities.

	Nothing		Few		Some		Much	
	*n*	%	*n*	%	*n*	%	*n*	%
***Academic aspects***								
Item 1. You have been sent materials, assignments, and homework by your School	6	1.2	8	1.6	146	29.2	340	68
Item 2. You spend enough time on your schoolwork during the day	4	0.8	43	8.6	249	19.8	204	40.8
Item 20. You work on school projects with your family throughout the day	184	36.8	172	34.4	118	23.6	26	5.2
***Routine***								
Item 3. You have an agreed routine and you try to stick to it	21	4.2	66	13.2	245	49	168	33.6
Item 4. You usually have breakfast, lunch, and dinner at the same time each day	26	5.2	68	13.6	221	44.2	185	27
Item 7. You have healthy sleeping habits	33	6.6	123	24.6	220	44	124	24.8
Item 13. You are eating a well-balanced diet	24	4.8	74	14.8	280	56	122	24
***Physical activity***								
Item 5. You get enough physical exercise during the day	45	9.0	213	42.6	183	36.6	59	11.8
Item 6. You move body (around) enough	17	3.4	202	40.4	211	42.2	70	14.0
***Emotions***								
Item 8. You cry more than usual	43	8.6	74	14.8	134	26.8	249	49.8
Item 9. You feel more nervous than usual	75	15.0	155	31.0	129	25.8	141	28.2
Item 10. You get angry more than usual	94	18.8	171	34.2	154	30.8	81	16.2
Item 11. You feel sadder than usual	55	11.0	114	22.8	168	33.6	163	32.6
Item 12. You are happy	18	3.6	131	26.2	290	58.0	61	12.2
***Addictions***								
Item 14. You are eating more than usual during lockdown	49	9.8	157	31.4	195	39.0	99	19.8
Item 15. You are eating more treats (e.g., cookies, chocolate, and chips) during lockdown	46	9.2	116	23.2	222	44.4	116	23.2
Item 16. You are over-using new technology	240	48.0	198	39.6	52	10.4	10	2.0
Item 17. You are watching too many TV programs, cartoons, or movies	111	22.2	209	41.8	134	26.8	46	9.2
***Playful and creative activities***								
Item 18 You are taking part in creative activities (e.g., theater, music, and art)	159	31.8	165	33.0	129	25.8	47	9.4
Item 19. You play different games throughout the day	36	7.2	200	40.0	205	41.0	59	11.8
Item 22. You play with your family throughout the day	184	36.8	172	34.4	118	23.6	26	5.2

With regard to the routine domain, the option of having experienced a much-agreed routine has been indicated by 33.6% (*n* = 168) of the adolescents, whereas 49% (n = 245) said that they have some routine. Likewise, 27% (*n* = 185) say that they eat breakfast, lunch, and dinner at the same time each day and 44.2% say that they do this sometimes (*n* = 221). Moreover, adolescents say that they have many habits (24.8%, *n* = 124) or some habits (44%, *n* = 220) of healthy sleeping and much (24%, *n* = 122) or some (56%, *n* = 280) balanced diet.

As regards the physical activity domain, 11.8% (*n* = 59) of the adolescents say that they do much exercise and 36.3% (*n* = 183) say they do some. In the same way, 14% (*n* = 70) say they move their body around much and 42.2% (*n* = 211) some.

With regard to the emotional domain, the majority of the adolescents think that they cry more than usual [49.8% said much (*n* = 249) and 26.8% said some (*n* = 134)]. 28.2% (*n* = 141) indicate that they are much more nervous and 25.8% (*n* = 129) indicate the option somewhat more nervous. 16.2% (*n* = 81) said that they get much more angry than usual and 30.8% (*n* = 154) indicate the option somewhat angrier. Likewise, adolescents feel much sadder 32.6% (*n* = 163) or somewhat sadder 33.6% (*n* = 168). However, some of them also say that they feel much happier 12.2% (*n* = 61) or somewhat happier 58.0% (*n* = 290).

With respect to the addictions domain, 19.8% (*n* = 99) think that they are eating much more than usual and 39% (*n* = 195) that they are eating some more. Moreover, adolescents are eating many more (23.2%; *n* = 116) or some more (44.4%; *n* = 222) treats and sweets. However, only 2.0% (*n* = 10) think that they are over-using the new technologies much and 10.4% (*n* = 52) that they are over using somewhat. In the same vein, only 9.2% (*n* = 46) think that they are watching too much TV and 26.8% (*n* = 134) that they are watching somewhat too many TV programs.

Finally, with regard to the playful and creative activities domain, the adolescents think that they are taking part in creative activities much (9.4%; *n* = 47) or some (25.8%; *n* = 129). Likewise, they think that they play different games throughout the day much (11.8%; *n* = 59) or some (41.0%; *n* = 205). However, only 5.2% of adolescents think that they play with their family much throughout the day 5.2% (*n* = 26) and 23.6% think that they play some (*n* = 118).

### The Well-Being of Adolescents in De-Escalation by Gender and Age

The results in Table 3 show that there are significant differences in the overall well-being, with boys obtaining higher scores *V* = 0.079[*F* (7,489) = 5.99, *p* = 0.001], η^2^ = 0.08]. In the same way, significant differences arise in relation with emotions, with boys having higher levels in the emotional domain (more positive emotions) [*F* (1,495) = 38.23, *p* = 0.001, η^2^ = 0.07] and addictions domain (fewer addictions) [*F* (1,495) = 5.61, *p* = 0.018, η^2^ = 0.011].

**TABLE 3:** Descriptive analysis and multivariate and univariate analysis of the different dimensions of well-being by sex and age.

Dimensions					
*Sex*		*n*	*M*	SD	
Emotions	Boy	174	3.05	0.62	
	Girl	323	2.66	0.72	
Playful and creative activities	Boy	174	2.20	0.54	
	Girl	323	2.17	0.65	
Academic	Boy	174	2.94	0.49	
	Girl	323	2.98	0.44	
Addictions	Boy	174	2.43	0.57	
	Girl	323	2.30	0.56	
Routine	Boy	174	3.09	0.54	
	Girl	323	2.99	0.54	
Physical activity	Boy	174	2.60	0.76	
	Girl	323	2.57	0.71	
Well-being Total	Boy	174	2.77	0.33	
	Girl	323	2.63	0.37	

**Age**		***n***	***M***	***SD***	

Emotions	12–14	110	2.93	0.65	
	15–16	200	2.84	0.73	
	17–18	186	2.64	0.70	
Playful and creative activities	12–14	100	2.41	0.62	
	15–16	200	2.20	0.59	
	17–18	186	2.03	0.53	
Academic	12–14	110	3.05	0.47	
	15–16	200	2.99	0.45	
	17–18	186	2.91	0.47	
Addictions	12–14	110	2.39	0.61	
	15–16	200	2.32	0.56	
	17–18	186	2.35	0.56	
Routine	12–14	110	3.17	0.53	
	15–16	200	3.01	0.60	
	17–18	186	2.95	0.57	
Physical activity	12–14	100	2.61	0.71	
	15–16	200	2.58	0.74	
	17–18	186	2.56	0.74	
Well-being Total	12–14	207	2.75	0.37	
	15–16	276	2.66	0.35	
	17–18	2.58	2.58	0.37	

***Sex***	***F***	***gl***	***p***	** η^2^**	

Emotions	38.24	1	0.001***	0.07	
Playful and creative activities	0.364	1	0.547	0.001	
Addictions	5.61	1	0.018*	0.01	
Academic	1.25	1	2.64	0.003	
Routine	2.94	1	0.087	0.006	
Physical activity	0.212	1	0.646	0.001	
Well-being Total	15.95	1	0.001***	0.03	

***Age***	***F***	***gl***	***p***	** η^2^**	***Post hoc***

Emotions	4.834	2	0.008**	0.02	1>3 3>1
Playful and creative activities	10.52	2	0.000***	0.04	1>3 2>3 3>1
Addictions	0.708	2	0.493	0.003	
Academic	1.686	2	0.187	0.007	
Routine	4.22	2	0.015**	0.02	
Physical activity	0.778	2	0.460	0.003	1>3;3>1
Well-being Total	7.72	2	0.001**	0.03	1>3;3>1

***p < 0.001.

**p < 0.01.

*p < 0.05.

On the other hand, taking into account the Multivariate Contrasts of Pillai’s Trace in relation to age, *V* = 0.081, [*F* (2,488), = 5.12, *p* = 0.001, η^2^ = 0.07], significant differences arise in the general well-being [*F* (2,493) = 7.72, *p* = 0.001, η^2^ = 0.03] with the younger adolescents (12–14 years) having higher scores. Similarly, differences arise in routine [*F* (2,493) = 4.22, *p* = 0.015, η^2^ = 0.02], emotion [*F* (2,493) = 4.83, *p* = 0.008, η^2^ = 0.02], and playful and creative activities [*F* (2,493) = 10.52, *p* = 0.001, η^2^ = 0.04], again with the younger ones having higher scores in all of these domains.

### Academic Task Stress and the Well-Being of the Adolescent

Table 4 shows the six well-being domains and the overall well-being of the participants and their correlation with the academic task stress. In fact, correlation exists between stress and all of the well-being domains in the total sample: emotions (*r* = 0.440), playful and creative activities (*r* = 0.214), addictions (*r* = 0.279), routine (*r* = 0.206), and physical activity (*r* = 0.238). In the same way, correlations exist between academic task stress and overall well-being (*r* = 0.334). In this sense, it could be said that the less stress the academic tasks caused to the participants, they feel more positive emotions, carry out more playful and creative activities, have less addictive behaviors, have more established routines and practice more physical activity. This table also shows the correlations of well-being controlling for sex and age of the sample.

**TABLE 4:** The correlations between academic task stress, well-being domains and the overall well-being, controlling for gender and age.

	Academic task stress-total sample	Boy	Girl	12–14	15–16	17–18
Emotions	0.440**	0.388**	0.457**	0.452**	0.446**	0.474**
Playful and creative activities	0.214**	0.043	0.190**	0.094	0.140	0.147
Addictions	0.279**	0.261**	0.292**	0.245**	0.344**	0.311**
Routine	0.206**	0.042	0.186**	0.151**	0.261**	0.003
Physical	0.238**	0.004	0.092	0.056	0.011	0.141
Academic	0.232**	0.315**	0.249**	0.311**	0.225**	0.272**
Overall well-being	0.334**	0.199**	0.369**	0.291**	0.400**	0.337**

**p < 0.01.

## Discussion

In this research, the well-being of adolescents in the pandemic situation was analyzed from a holistic perspective, taking into account different aspects such as physical, emotional, social and academic domains. Firstly, the results of the study point out that the general well-being of adolescents in a lockdown situation is situated at an intermediate level. Taking into account the different aspects measured within the general well-being, the domains that obtained the lowest scores were the addictions and playful and creative activities that they carry out; that is to say, teenagers tended to engage in addictive activities, and were less likely to engage in any playful or creative activities. Intermediate scores were also obtained in the emotional domain and the physical activity that young people practice, with the routine aspects and academic aspects (the perception that they have that they received sufficient school tasks and that they spend enough time doing these) having the highest scores.

As for the academic domain, one of the biggest concerns of the adolescents during the lockdown has been their schoolwork and the uncertainty about their academic future (Lee, 2020). In the present study, most adolescents have reported spending a lot of time doing academic work, though many have not received help from their parents. Although having done a lot of academic work may be favorable to their academic development, we must contemplate that adolescents have had to face this challenge alone. There are few parents who have helped or have been able to help their sons and daughters, either because they were working too (Roig and Nebot, 2020) or because they did not have the necessary academic level (Vivanco-Saraguro, 2020). Indeed, another fact to take into account is that schoolwork has been one of the causes of conflict in families during lockdown (Burgess and Sievertsen, 2020; Cluver et al., 2020; Idoiaga et al., 2020). In fact, the involvement of parents in helping their children academically and taking care of relationships could help not only in their performance, but also in the development of the adolescents’ self-concept, an important aspect to foster during adolescence (Álvarez et al., 2015). Therefore, the state of lockdown has not been the most favorable for the education of adolescents and has had negative effects on educational aspects (Abad, 2020).

Regarding the routine domain, not having face-to-face classes could alter adolescents’ routines, as indicated by some authors (Young Minds, 2020). However, in the present study, the adolescents reported having good routines and having maintained the appropriate breakfast/lunch/dinner routines. They have also had a good quality of sleep, which is essential for taking care of their physical, psychological, and cognitive health. Sleep has a repairing effect and helps to reinforce the memory, which is essential for their academic performance (Rasch and Born, 2013; Troynikov et al., 2018. Therefore, despite the fact that the adolescents have been in the harsh conditions of lockdown, they have been able to maintain routines and this has been a favorable outcome for their well-being.

In contrast to other studies that reported that confinement brought decreased physical activity and sedentary behavior among adolescents (Ammar et al., 2020; Ruíz-Roso et al., 2020), many adolescents in the sample reported getting plenty of exercise. This is an aspect that plays in the adolescents’ favor, since 150–300 min of exercise per week has been recommended during the pandemic (Piercy et al., 2018) as some data indicate that exercise may reduce the risk of acute respiratory distress syndrome, per COVID-19 (University of Virginia Health System, 2020). Sport has been able to help adolescents not only to stay physically healthy, but also to manage the stress of the pandemic and avoid other bad habits, such as alleviating stress with food or other substances (Nyenhuis et al., 2020). In addition, it has been proven that sports have a favorable influence on academic performance (Bradley et al., 2013).

However, most adolescents have admitted to eating more during lockdown, and mostly to eating more unhealthy food. Beyond the risks that the increase in the rate of obesity may pose in the long term, it should also be taken into account that an optimal nutritional status is essential during a pandemic to strengthen the immune system (Iddir et al., 2020). Poor eating habits acquired during the lockdown may also have influenced their education because poor diet has been shown to lower academic performance (Florence et al., 2008).

One of the most surprising results of this research is the low number of adolescents who admit to having abused new technologies or screens. In itself, this seems a positive aspect, but it is very striking because all indications and research worldwide point to the fact that new technologies and screens have been abused during lockdown (Montag and Elhai, 2020). We should therefore ask ourselves and analyze whether in fact adolescents are aware of this abuse or have framed it as something normal since they are already prone to spending more time than adults using ICTs (Salaway et al., 2008). In fact, if young people do not have the capacity to see or admit to a problem concerning the overuse of technologies, the problem of this addiction would be aggravated and urgent strategic plans for training and awareness would be necessary.

In addition, the abuse of technology may also arise from the difficulty of managing stress created by the pandemic. The emotional and academic difficulties of adolescents are more likely to be a cause of the abusive use of technology (de la Villa Moral and Suárez, 2016; Hawi and Samaha, 2016; Sert et al., 2019) and it is therefore important to pay attention to the emotional care of adolescents during lockdown and restrictive periods.

In the emotional domain, from the beginning of the pandemic, adolescents have been cataloged as an at-risk population (The Lancet Child Adolescent Health, 2020). In the present study, they have admitted to being more nervous and sadder than usual, maybe because of being away from their peers, which is very important for their age-group (Meeus and Dekoviíc, 1995). In fact, adolescence is a time of transition when young people may already be experiencing difficult emotional moments (Imran et al., 2020). It is therefore important to promote their well-being by encouraging them to remain calm and take measures to reduce stress (Guessoum et al., 2020). Indeed, the World Health Organization published recommendations addressed to adolescents from the beginning of the pandemic to help them cope with stress (WHO, 2020).

Regarding the playful and creative activities, teenagers are not feeling very creative or playing much with the family. This may be due to the fact that, in adolescence, play is not like in the childhood stage and adolescents start to interact more with their peers (Meeus and Dekoviíc, 1995) and not feel so comfortable with parents. Adolescents attach more importance to their peers than to the family at this stage, which is why they may be feeling high levels of loneliness and boredom (Oxford ARC Study, 2020; Young Minds, 2020).

As far as gender differences in adolescents are concerned, the results show that boys have expressed more emotions than girls in the present study. In fact, it has been observed that boys have a greater positive emotional expression. These results contradict studies that say that girls tend to be more likely to express positive emotions (Brody, 1999; Chaplin and Aldao, 2013). This may be due to the fact that women have shown less well-being than men in this study and therefore express less positive emotions. In fact, there is often a tendency for girls to internalize feelings of distress, which may increase their propensity for depression (Zahn-Waxler et al., 2008).

With respect to age differences, it is the younger adolescents who show the greatest well-being compared to the older ones. This may be due, among other things, to the fact that they do not yet have the same pressure from academic staff as the older ones who are finishing their studies of secondary education. In fact, the schooling of adolescents and having to study at a distance has also been one of the greatest challenges of this pandemic and one of the main stressors for families (Burgess and Sievertsen, 2020; Cluver et al., 2020). In addition, the fact that older adolescents do more academic work, due to the demands that the last stage of secondary education brings, could lead them to have less healthy habits and take less care of their eating and sleeping routines. That is, older students may be experiencing anxiety about university entrance exams (Sari et al., 2018), and therefore have less healthy habits and not take care of routines. It may moreover be because they have less free time that older adolescents also practice creative activities to a lesser degree.

Finally, with regard to the academic task stress, results have indicated that this correlates with the overall well-being of the adolescents, and also with some of the different analyzed domains (routine, physical exercise, additions, academic aspects, and creative and playful activities). The emotional domain is the one that show the highest negative correlation with academic stress, that is to say, the more stressed the teenagers were, the less emotional well-being they had. Likewise, from these results, we can also conclude that having a greater well-being and taking care of the physical, emotional, playful and routine domains may be also important for preventing the stress of academic tasks. In addition, it is known that stress has a negative influence on academic performance (Arsenio and Loria, 2014), so it is important to take care of this aspect during lockdown or emergency e-learning periods to improve the education of adolescents.

Therefore, from a holistic perspective of adolescent well-being, the results of this research have shown that the overall well-being of adolescents in lockdown is at an intermediate level. This may be due to a balance between different dimensions. That is, the low levels of well-being related to addictions (mostly for screen use and eating behaviors) and playful activities, the intermediate levels created by ambibalent emotions and physical activity, and the high levels of routine and academic aspects make for an intermediate level of total well-being. These results are not critical but should be viewed with caution and followed up as the pandemic period does not end with lockdown and restrictions for children and adolescents are still in place in many countries.Therefore, it would be important to carry out similar cross-sectional studies.

Regarding the limitations of the study, the main limitations are related to the measurement instruments used. The questionnaire used to assess well-being is validated in a population between 2 and 14 years of age; however, the study sample is between 12 and 18 years of age, and the questionnaire has been supplied in this population with good internal consistency and reliability. Likewise, the instrument used to assess the stress of the academic task during confinement is *ad hoc* perhaps for further analysis will be interesting to add other validated scales to measure this. Therefore, the analyses performed with these questions should be interpreted with caution.

Moreover, the generalizability of the results is limited, since it is a non-probabilistic sample in which there may be a certain selection bias. That is, participation was voluntary, and the respondents may well have been adolescents who were especially emotionally impacted by the pandemic. Future studies should expand the sample and extend it to more countries.

## Conclusion

In conclusion, the results show that the pandemic situation and the different measures that the government is taking to protect the health of the population (such as the lockdown or restrictive periods) are generating a direct effect on adolescents. For this reason, it is important to take care of this vulnerable collective by attending to their well-being and promoting quality socio-educational policies based on the needs that have been identified in this study. To accompany adolescents in their educational processes, to offer them spaces to carry out physical activities, to promote habits that safeguard their routines, to encourage them to participate in creative and playful activities, to guarantee that they continue to maintain relationships with their peers, to give them tools to be able to face this new situation emotionally and to control the excessive consumption of food and use of screens are therefore some of the key factors that we must address in the near future.

## Data Availability Statement

The raw data supporting the conclusions of this article will be made available by the authors, without undue reservation.

## Ethics Statement

The studies involving human participants were reviewed and approved by University of the Basque Country. Written informed consent to participate in this study was provided by the participants’ legal guardian/next of kin.

## Author Contributions

NB and MD-S were involved in the and analysis of the data. NI and N-OE were involved in the interpretation of the data. All authors were involved in the conceptualization of the project, acquisition of the data, drafting and revising of the work for intellectual content, provided approval for submission of the contents for publication, and agreed to be accountable for the accuracy and integrity of the project.

## Conflict of Interest

The authors declare that the research was conducted in the absence of any commercial or financial relationships that could be construed as a potential conflict of interest.

## Publisher’s Note

All claims expressed in this article are solely those of the authors and do not necessarily represent those of their affiliated organizations, or those of the publisher, the editors and the reviewers. Any product that may be evaluated in this article, or claim that may be made by its manufacturer, is not guaranteed or endorsed by the publisher.
